# Supplementary material for: Complexation with Polysaccharides Enhances the Stability of Isolated Anthocyanins
Source: Foods. 2023 Apr 29;12(9):1846. doi: 10.3390/foods12091846 (PMC10178255; doi:10.3390/foods12091846)
Supplement: Supplementary file 1 [file foods-12-01846-s001.zip › foods-2287239-supplementary.pdf]

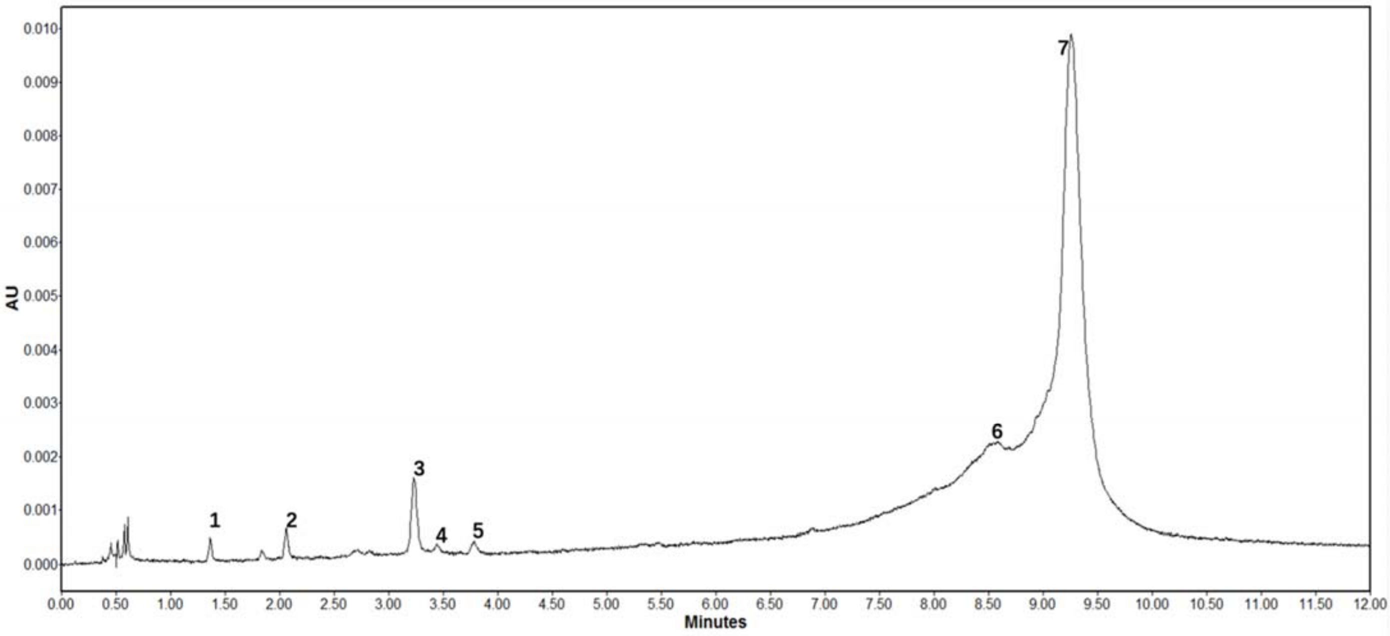

Figure S1. Illustrative UPLC chromatogram of the anthocyanin fraction from purple potatoes. Peak assignment: 1. gallic acid; 2. protocatechuic acid; 3. chlorogenic acid; 4. caffeic acid; 5. p-coumaric acid; 6. malvidin chloride; 7. petunidin chloride.

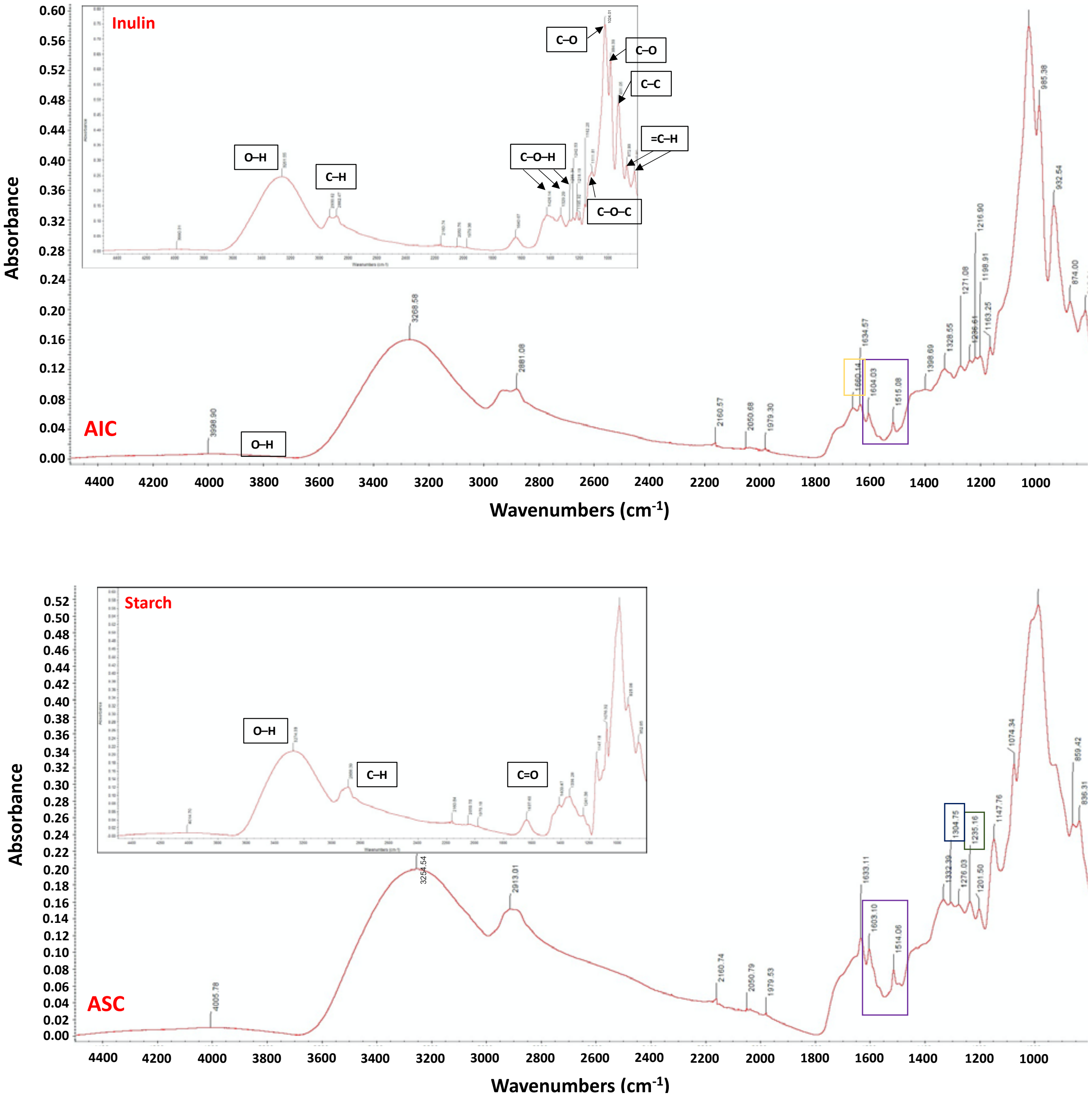
Inulin
O–H
C–H
C–O–H
C–O
C–O
C–C
=C–H
C–O–C
AIC
O–H
Absorbance
Wavenumbers (cm-1)
3268.58
2881.08
3998.90
2160.57
2050.68
1979.30
1660.14
1634.57
1604.03
1515.08
1398.69
1328.55
1271.08
1236.61
1216.90
1198.91
1163.25
985.38
932.54
874.00
Starch
O–H
C–H
C=O
ASC
3254.54
2913.01
4005.78
2160.74
2050.79
1979.53
1633.11
1603.10
1514.06
1332.39
1304.75
1276.03
1235.16
1201.50
1147.76
1074.34
859.42
836.31

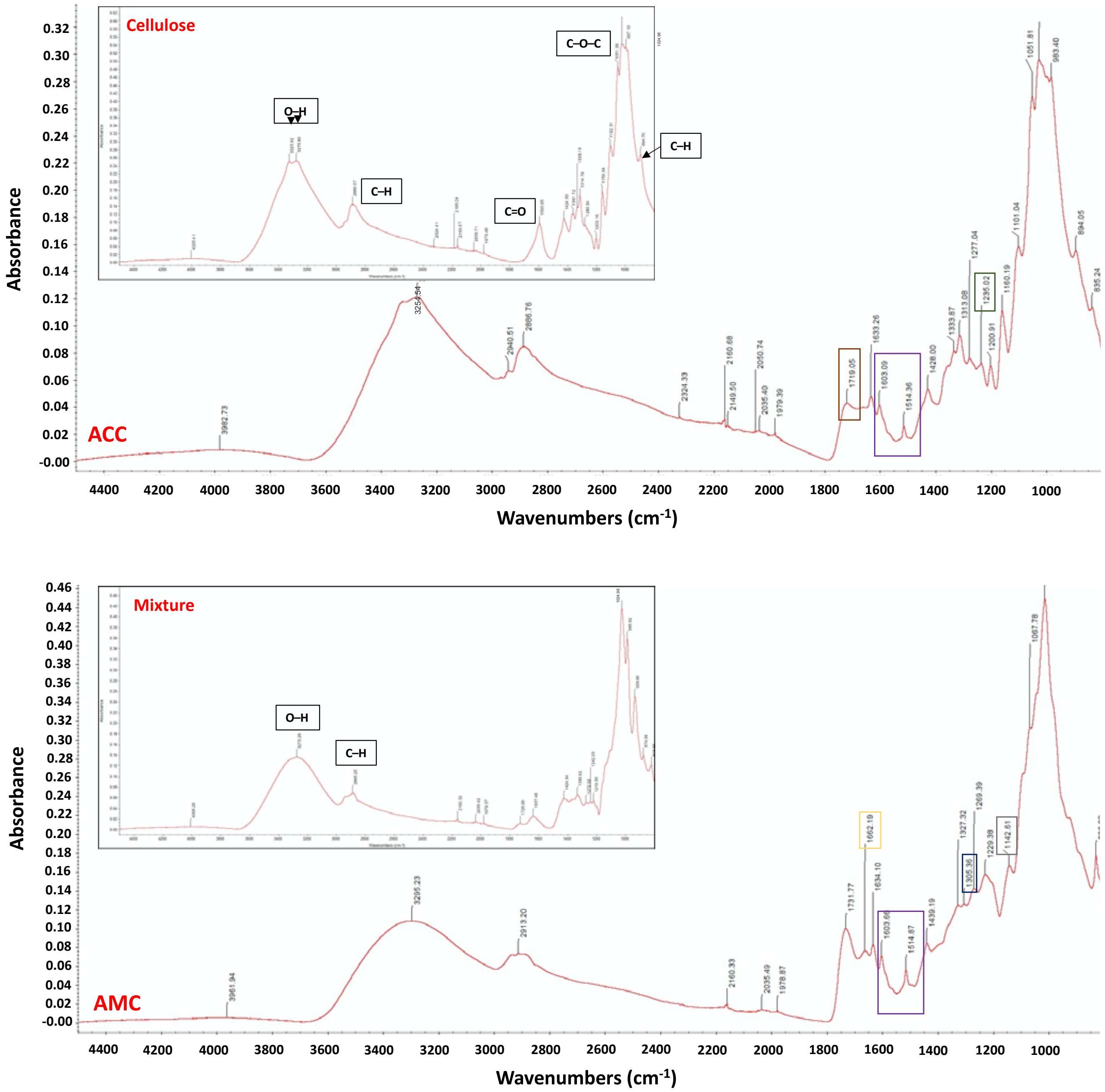

Figure S2. FTIR spectra. AIC, anthocyanin-inulin complex; ASC, anthocyanin-starch complex; ACC, anthocyanin-cellulose complex; AMC, anthocyanin mixture of polysaccharides complex.
